# Prevalence Rates and Evolution of Psychiatric Disorders Among Incarcerated Youths in Comparison With Non-incarcerated Youths

**DOI:** 10.3389/fpsyt.2021.784954

**Published:** 2022-01-07

**Authors:** Patrick Heller, Larisa Morosan, Deborah Badoud, Manon Laubscher, Lisa Jimenez Olariaga, Martin Debbané, Hans Wolff, Stéphanie Baggio

**Affiliations:** ^1^Division of Prison Health, Geneva University Hospitals, University of Geneva, Geneva, Switzerland; ^2^Department of Psychiatry, University of Geneva, Geneva, Switzerland; ^3^Faculty of Psychology and Educational Sciences, University of Geneva, Geneva, Switzerland; ^4^Institute of Primary Health Care (BIHAM), University of Bern, Bern, Switzerland

**Keywords:** prison, juvenile, delinquency, psychiatry, mental health

## Abstract

**Background:** Our main objective was to provide estimates of the prevalence rates of psychiatric disorders and comorbidities among youths in a juvenile detention center in Geneva, Switzerland. We also aimed to investigate potential positive effects of intensive psychotherapeutic and educational services this center provides. Finally, we examined psychiatric care prior to and after custody as well as the evolution of the youths' mental health during detention.

**Methods:** We conducted a longitudinal study including a group of incarcerated (*n* = 86) and a group of non-incarcerated (*n* = 169) youths (12–18 years old). Measures included diagnoses of psychiatric disorders, cognitive functions, trauma, psychopathic traits and the Youth Self-Report (aggressive behavior, attentional disorders, criminal behavior, social withdrawal, anxiety, depression and somatic complaints) collected at baseline and at discharge for the incarcerated group. Data were analyzed using mixed-effect models.

**Results:** Psychiatric disorders were prevalent in the incarcerated group (82.6, 95% CI: 71.6–90.7%), but young people also often suffered from several disorders simultaneously. Two-thirds of the incarcerated participants had a diagnosis of two or more psychiatric disorders. Regarding health care, most incarcerated participants (79.1%) had psychiatric care prior to detention. The planned care after detention was associated with psychiatric comorbidities, care being more likely planned for those with comorbidities (*p* = 0.030). Compared to the non-incarcerated group, the incarcerated group had lower scores on cognitive functioning (*p* < 0.001) and higher scores on trauma (*p* < 0.021) and psychopathic traits (*p* < 0.034). The youths' stay in the detention center was associated with a positive change of mental health, with externalized problems being significantly reduced at the end of their stay (*p* = 0.017).

**Conclusion:** Our findings showed that youths in conflict with the law are characterized by (1) their internal vulnerabilities: a high prevalence of psychiatric disorders and psychiatric comorbidities, lower cognitive functions, externalized problems and psychopathic traits; (2) environmental factors: victims of violence and sexual abuse; and (3) their psychiatric history. Besides, the evolution of the most prevalent issues was favorable over time, which puts into question the usual perspective about the deleterious effect of detention.

## Introduction

Criminology has provided a growing understanding of the functioning of juvenile delinquency and its risk factors during the twenty-first century, providing a picture of the care this population needs (1–3). However, interest in categorical psychiatric diagnoses and predictive factors of recidivism in youths in conflict with the law only began at the end of the twentieth century. Currently, several epidemiological studies have highlighted the high prevalence rate of psychiatric disorders among incarcerated minors. This prevalence rate ranges between 40 and 90% (4–9). The prevalence rate is higher for most psychiatric disease among female youths (10).

A literature review shows that 69.9% of male youths in custody had at least one psychiatric disorder, most commonly conduct disorder, substance use disorders and attention deficit hyperactivity disorder [ADHD; (11)]. The high prevalence rate of ADHD among incarcerated juveniles is 3–5 times higher (30%) than in the general population. This has detrimental effects on behavioral disorders in detention as well as on the risk of recidivism and issues related to reintegration (12, 13). Such high prevalence rates of pre-existing psychological disorders and comorbidities require specialized care.

However, access to psychiatric and psychotherapeutic care in custody remains insufficient, given the high prevalence rate of psychiatric disorders and the crucial need of mental health care for these young populations (14, 15). Furthermore, major psychiatric disorders are commonly associated with criminal behavior (16, 17). Providing youth with mental health treatment can effectively reduce recidivism, although the factors associated with treatment effectiveness are difficult to identify. Indeed, there is a lack of studies that examine the effectiveness of custodial intervention programs (18). The prison management can also try to reduce young prisoners' mental health problems through developing scientific procedures for their mental health assessment and creating a correctional climate that is more beneficial (19). Studies have found that intensive psychotherapeutic care during custody reduces the risk of recidivism upon release (20, 21). Overall, the effects of custody do not have a positive effect on the mental health of incarcerated youth, mainly because of the lack of psychiatric and educational care during incarceration (22).

Therefore, identifying mental health disorders is essential to prevent recidivism. In Switzerland, outpatient treatment measures that the juvenile court ordered increased by more than 300% between 1999 and 2015, suggesting that juvenile judges have become more sensitive to minors' mental health (23).

Apart from epidemiological and mental care aspects, an important research area is the relationship between risk factors and delinquency/recidivism. Intrapersonal (e.g., cognitive, emotional, psychiatric and neurological) and environmental (e.g., history of family violence and abuse) vulnerabilities coexist in delinquent youths and improve the understanding and prediction of violent crime in adulthood (2). For example, adverse childhood experiences have been extensively studied in psychology and criminology (24–27), representing an important risk factor for recidivism (26, 27). Trauma children experience does not only have a direct effect on the risk of recidivism, but also an indirect effect through developing psychiatric disorders (28). However, the risk of recidivism depends on the severity and repetition of the traumas. The greater they are, the less effective protective factors, such as social ties, will be (29).

The objectives of this study were (1) to provide estimates of the prevalence rates of psychiatric disorders and comorbidities among youths in a detention center for minors in Geneva, Switzerland; (2) to examine psychiatric care prior to and after custody for this group of youths and the evolution of their mental health during detention, in comparison with a group of non-incarcerated youths; and (3) to investigate potential positive effects of intensive psychotherapeutic and educational care.

## Materials and Methods

### Setting and Participants

This observational longitudinal study included two groups of participants. Eighty-six youths admitted to the observation sector of a juvenile detention center in Geneva participated to the study between December 2011 and May 2018 (incarcerated group, mean age = 15.96, 29.1% girls, see Table 1 for detailed descriptive statistics). The study also included 160 youths from the general population (non-incarcerated group, mean age = 15.81, 49.4% girls, see Table 1). The non-incarcerated group was a convenient sample. The incarcerated group was assessed twice (median = 41.5 days, range: 7–193 days): at the beginning and the end of the detention period. Only 54 youths accepted to be tested at the end of their detention period. The inclusion criteria were being between 12 and 18 years of age and having a good knowledge of French. The youths admitted to the detention center represented a subgroup of all minors in conflict with the law who required intensive interdisciplinary care in a secure and medicalized environment. Youths who participated in the second assessment were not significantly different from those who only participated in the baseline assessment (sociodemographic and clinical variables).

**Table 1:** Descriptive statistics for the incarcerated and non-incarcerated groups.

	**Group**	
	**Incarcerated**	**Non-incarcerated**
*n*	86	160
**Age** (mean, standard deviation)	15.96 (1.20)	15.81 (1.81)
Age min–max	12.75–18.16	12.01–19.07
**Gender** (%, *n*)		
Male	70.9 (61)	50.6 (81)
Female	29.1 (25)	49.4 (79)
**Socioeconomic level** (%, *n*)		
Low	57.3 (47)	31.7 (44)
Intermediate or high	42.7 (35)	68.3 (95)
**Psychiatric care before detention** (%, *n*)		
Yes	79.1 (68)	–
None known	20.9 (18)	–
**Reason for admission** (%, *n*)		
Civil placement—absence of offense	23.3 (20)	–
Minor offences	55.8 (48)	–
Crimes	20.9 (18)	–
Length of stay (median)	118 days	–
Duration of stay Min-max	54–473	–
**Criminal psychiatric expertise** (%, n)		
Yes	17.4 (15)	-
No	82.6 (71)	-
**Post-detention accommodation** (%, *n*)		
Parents' home	38.4 (33)	-
Open educational home	50.0 (43)	-
Closed educational home	11.6 (10)	-
**Psychiatric care after detention** (%, *n*)		
Planned	72.1 (62)	-
Not planned	27.9 (24)	-

The head psychiatrist (PH) recruited the incarcerated participants and they participated in the study in the detention center's dedicated facilities. Youths in the “non-incarcerated” group were recruited through posting flyers at the university, in different afterschool activities and through word of mouth. The interviews took place at the University of Geneva. Trained psychologists and research assistants conducted the interviews. The participation in the study was voluntary and informed consent was obtained from the participants and their legal guardians. A music shop voucher worth 50 CHF was rewarded to the participants in both groups. The research protocol was approved by the ethics commission of the Department of Psychiatry of the Faculty of Medicine of the University of Geneva (no. 2010-10-240).

### Measures

#### Diagnoses of Psychiatric Disorders

Psychiatric disorders were assessed according to *DSM-IV* criteria using the semi-structured assessment tool, Kiddie-SADS Present and Lifetime Version (K-SADS-PL) (30). Psychiatric disorders present throughout life (past and present) were considered.

#### Cognitive Functions

We assessed cognitive functions using the 4th French version of the Wechsler Intelligence Scale for Children (WISC) (31). Only four subtests were used to reduce the length of the assessment. The vocabulary subtest assessed lexical stock, language development and concept understanding; the digit span subtest assessed short-term memory; the block design subtest assessed visuo-constructive and visuo-spatial analysis abilities and the information subtest explored general knowledge. We chose these subtests because they cover a wide variety of cognitive processes generally described in the literature on juvenile offenders as verbal and performance abilities (32, 33).

#### Self-Assessment of Psychological Problems

We used the French version of the Youth Self Report (YSR) (34, 35) to assess externalized (aggression and rule breaking) and internalized (social withdrawal, anxiety, depression and somatic complaints) problems over the previous 6 months. The questionnaire contains 119 items with three response choices: 0 (not true), 1 (sometimes true) and 2 (often true). We used the three global scales of the YSR: externalized problems (total of the subscales of rule violation and aggressive behavior), internalized problems (total of the subscales of anxiety/depression, withdrawal/depression and somatic complaints) and total problems (total of the previous subscales as well as thought, attention and social problems).

#### Trauma

The Childhood Trauma Questionnaire (CTQ) is a screening inventory that assesses self-reported experiences of abuse and neglect in childhood and adolescence. It includes 70 items with a Likert-type scale consisting of five response choices (from 1 = “never true” to 5 = “very often true”). The CTQ is composed of 5 subscales and propose cut-offs to qualify the results as clinically significant (emotional abuse = 9, physical abuse = 8, sexual abuse = 6, emotional neglect = 10 and physical neglect = 8) (36).

#### Psychopathy

We used the Youth Psychopathic Inventory (YPI) (37) to examine psychopathic traits. The questionnaire has 50 items that assess three dimensions of psychopathy, each consisting of several subscales: (1) an interpersonal dimension that assesses dishonest charm, grandiosity, tendency to lie and manipulative behavior; (2) an affective dimension that assesses callous unemotional traits; and (3) a dimension assessing impulsivity, sensation-seeking and irresponsible behavior. The 50 YPI items are assessed on a scale of 1 (not applicable at all) to 4 (fully applicable).

#### Socio-Demographic and Life Trajectory Variables

These variables included age, gender, socio-economic level (low vs. intermediate or high), reason for admission in the detention center (civil placement: placement for purposes of assistance the civil court ordered due to serious endangerment, such as running away, prostitution, recurrent toxic consumption, refusal to adhere to the medico-educational devices set up; minor offenses; crimes: murder, serious bodily harm, rape, robbery, hostage taking, endangering the life of others), psychiatric care before detention (yes or not known), criminal psychiatric expertise by an external expert per the Juvenile Court's decision during incarceration (yes or no), duration of the stay, post-detention accommodation (parents' home, open educational home, closed educational home) and psychiatric care after detention (planned or unplanned).

We collected the K-SADS-PL and life trajectory variables (socio-economic level, reason for admission in the detention center, psychiatric care before detention, criminal psychiatric expertise, duration of the stay) only for the incarcerated group at the beginning of the study (*n* = 69 for K-SADS-PL). We collected post-detention accommodation and psychiatric care after detention at the end of the stay for the whole sample of the incarcerated group. Finally, we collected the YSR twice for participants in the incarcerated group, at baseline and at discharge (*n* = 54).

### Statistical Analyses

No sample size was computed *a priori*. We computed a sensitivity power analysis to assess the minimum effect size the study could detect. With *n* = 86 in the incarcerated group, *n* = 160 in the non-incarcerated group, alpha = 0.05, power = 0.80, and a two-tailed independent t-test, the effect size was *d* = 0.38. Therefore, our study identified moderate effect sizes.

We used descriptive analyses first to characterize participants, using percentages and means by type of variables. Four sets of analyses were then conducted to answer the research questions of the study. (1) We computed the prevalence rates of psychiatric disorders and comorbidities in the incarcerated group, including 95% confidence intervals. (2) We explored the relationship between diagnosed psychiatric disorders, presence of comorbidities and pre- and post-release psychiatric care using simple logistic regressions. (3) We compared cognitive function, self-reported psychological problems (YSR), trauma (CTQ) and psychopathy (YPI) in the incarcerated and non-incarcerated groups using simple and multiple linear regressions (adjusting for age, gender and socio-economic background). Effect sizes (Cohen's d) were computed for all statistically significant comparisons, with *d* = 0.2 considered as a small effect size, *d* = 0.5 moderate and *d* = 0.8 strong (38). (4) Finally, we explored changes during the stay in the level of the incarcerated group's psychological problems using the two measures of self-reported psychiatric problems (YSR) using linear mixed models, unadjusted and then adjusted for age, gender, socio-economic background and the number of days in detention before the youths were included in the study.

Analyses were performed using Stata 16 and G^*^Power 3.1.

## Results

### Prevalence Rates of Mental Disorders, Comorbidities and Psychiatric Care in the Incarcerated Group

**Figure 1:**
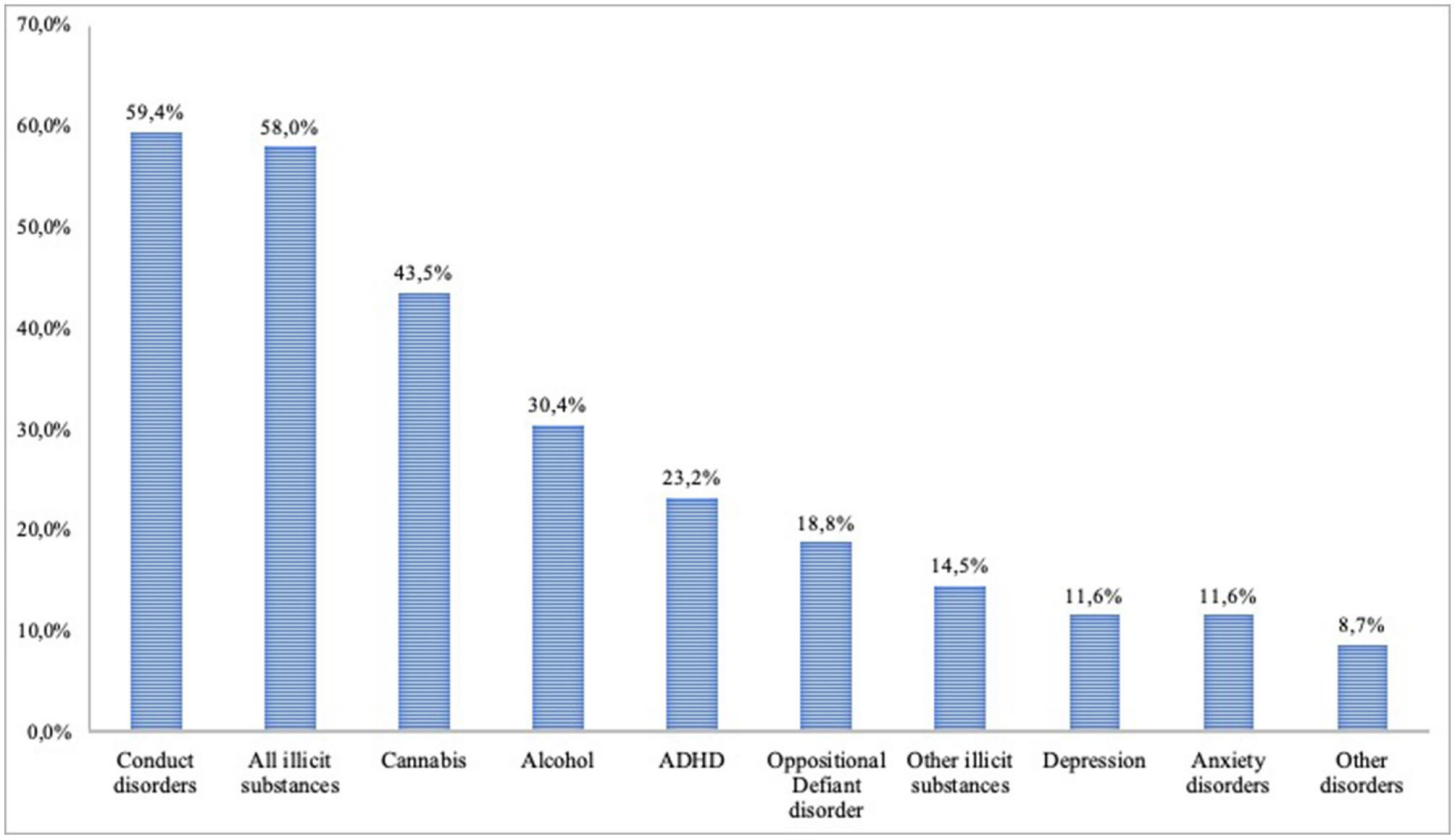
Prevalence rates of DSM-IV psychiatric diagnoses in incarcerated group. ADHD: attention deficit/hyperactivity disorder. Anxiety disorders: panic disorder, separation anxiety, hyper-anxiety, simple phobia, agoraphobia, obsessive-compulsive disorder, post-traumatic stress disorder. Other disorders: adjustment disorder with depressed mood, adjustment disorder with conduct disorder, bulimia, enuresis, hypomania, mania. Substance Use Disorders: includes abuse and dependence.

Overall, 82.6% of participants had at least one psychiatric disorder diagnosed using K-SADS-PL (95% CI: 71.6–90.7%). The most common types of disorders were substance use disorders (62.3, 05% CI: 50.2–73.1%). More specific, the prevalence rate was 58.0% for illicit substance use disorders (95% CI: 45.5–69.8%), 30.4% for alcohol use disorder (95% CI: 19.9–42.7%), 43.5% for cannabis use disorder (95% CI: 31.6–56.0%) and 14.5% for other substances (95% CI: 7.2–25.0%). The prevalence rate of having any externalized disorder was 26.1% (95% CI: 17.0–37.9%), including conduct disorders (59.4, 95% CI: 46.9–71.1%), ADHD (23.2, 95% CI: 13.9–34.9%) and opposition/provocative disorder (18.8, 95% CI: 10.4–30.1%). The prevalence rate of having any internalizing disorder was 18.8% (05% CI: 11.2–30.0%), including depression and anxiety (both: 11.6, 95% CI: 5.1–21.6%). Finally, 8.7% of participants had other disorders (95% CI: 3.3–18.0%). The detailed prevalence rates are presented in Figure 1.

**Figure 2:**
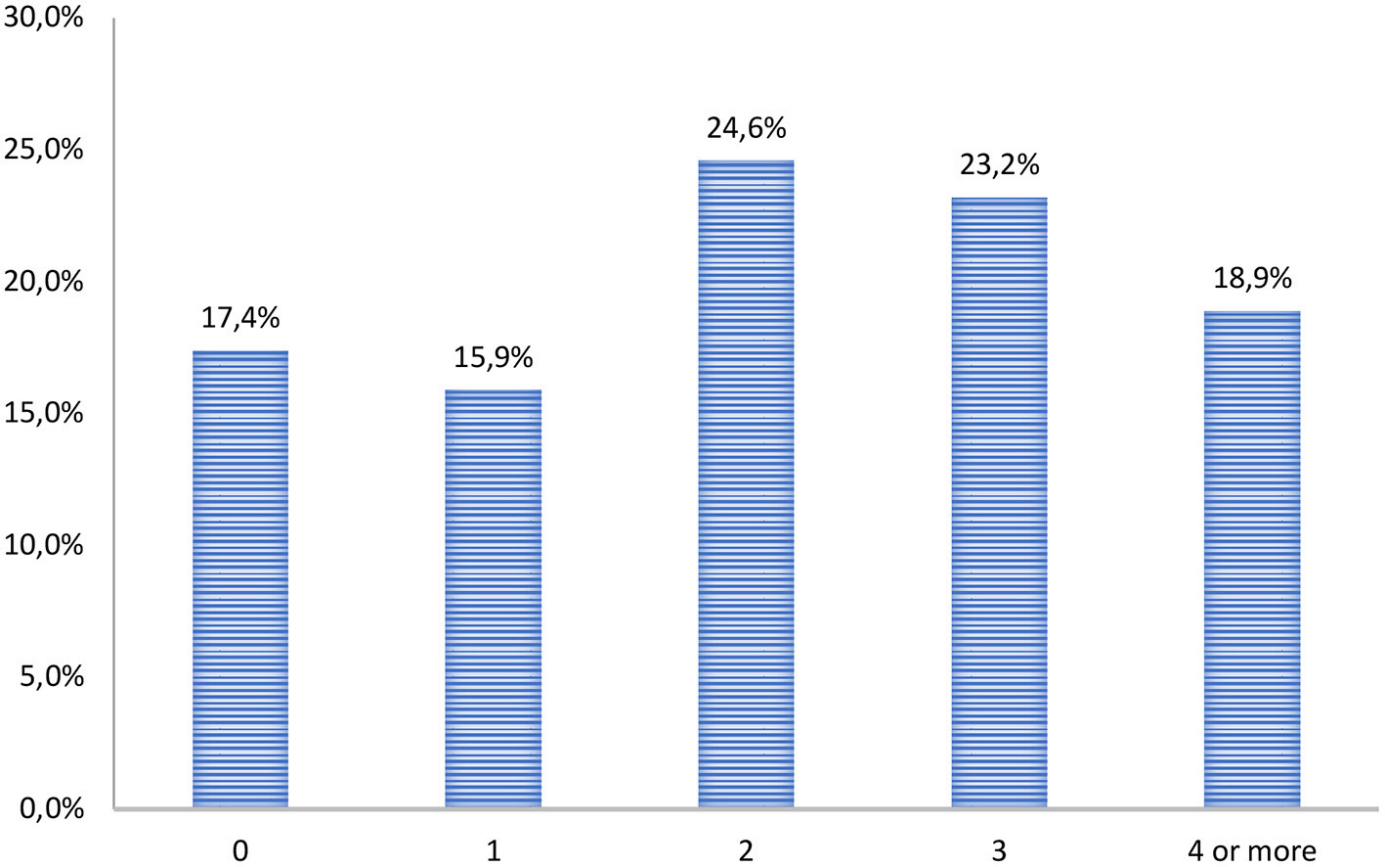
Number of psychiatric comorbid disorders in incarcerated group.

Participants most often presented more than one psychiatric disorder, as shown in Figure 2: 66.7% had two or more psychiatric disorders diagnosed using K-SADS-PL. Only 17.4% of participants had no diagnosed psychiatric disorder.

In addition, we explored the differences in pre- and post-release psychiatric care according to the presence of at least one psychiatric disorder and the number of comorbidities (Table 2). The results showed that having at least one psychiatric diagnosis was not related to psychiatric care, neither before (*p* = 0.732) nor after (*p* = 0.974) detention. Similarly, the number of psychiatric comorbidities was not significantly related to pre-detention psychiatric care (*p* = 0.665). Conversely, the more psychiatric comorbidities participants had, the more likely they had a planned psychiatric care after detention (*p* = 0.030).

**Table 2:** Pre- and post-release psychiatric follow-up according psychiatric disorders diagnosis in incarcerated group.

	**At least one psychiatric disorder diagnosis**		**Psychiatric comorbidities diagnosis**	
**Psychiatric care**	**Percentage**	***p*** **-value^a^**	**Number (0–5)**	***p*** **-value^b^**
**Pre-detention**				
None known	85.7 %	0.732	1.64 (1.15)	0.665
Yes	81.8 %		1.82 (1.39)	
**Post-detention**				
Not planned	82.4 %	0.974	1.18 (0.81)	0.030
Planned	82.7 %		1.98 (1.42)	

a *Simple logistic regressions*,

b *Simple ANOVAs*.

### Comparisons of the Incarcerated and Non-incarcerated Groups

Table 3 presents the comparisons between groups. Cognitive functions were lower in the incarcerated group, with significantly lower scores for all subtests (*p* < 0.001). Nevertheless, effect sizes were moderate for the digit span and block design subtests while they were strong for the vocabulary and information subtests.

**Table 3:** Comparisons between incarcerated and non-incarcerated groups.

	**Incarcerated (*n =* 86)**	**Non-incarcerated** **(***n =*** 160)**	**unadjusted p-value^a^**	**adjusted p-value^b^**	**Clinical threshold**	**Threshold limit**
**Cognitive functions**						
Vocabulary	7.51	10.83	**<0.001**	**<0.001**	–	–
Memory	8.16	9.54	**<0.001**	**<0.001**	–	–
Cube	8.18	9.91	**<0.001**	**<0.001**	–	–
Information	7.20	10.56	**<0.001**	**<0.001**	–	–
**Global scales youth self report**						
Externalized problems	70.57	56.97	**<0.001**	**<0.001**	63	60
Internalized problems	53.69	53.11	0.669	0.339	63	60
Total problems	63.11	56.25	**<0.001**	**<0.001**	63	60
**Childhood trauma questionnaire**						
Emotional abuse	8.64	7.49	**0.040**	**0.008**	9	–
Physical abuse	9.13	5.89	**<0.001**	**<0.001**	8	–
Sexual abuse	6.54	5.31	**<0.001**	**<0.001**	6	–
Emotional Neglect	11.94	10.81	0.096	**0.021**	10	–
Physical neglect	8.08	6.78	**0.001**	**0.001**	8	–
**Youth psychopathy traits inventory**						
Lifestyle	14.71	11.99	**<0.001**	**<0.001**	–	–
Interpersonal relations	9.99	9.05	**0.014**	**0.034**	–	–
Affectivity	10.81	9.40	**<0.001**	**<0.001**	–	–

*Internalized problems: total anxiety/depression, withdrawal/depression, somatic complaints*.

*Externalized problems: total violation of rules, aggressive behavior*.

*Total problems: total of the internalized, externalized, attention, social and thinking problem scales*.

a *p-value simple linear regression*.

b *p-value multiple linear regression controlling for age, gender, and socio-economic level*.

*Highlighted in bold: statistically significant differences at the 0.05 cut-off between the two groups*.

Comparisons of self-reported psychiatric problems between groups showed that participants in the incarcerated group scored significantly higher on all problems except the YSR internalizing problems (*p* = 0.339). Overall, participants in the incarcerated group had more externalized problems (*p* < 0.001) and a higher total of problems (*p* < 0.001) compared to what the participants in the non-incarcerated group had. The effect size was large for externalized problems and total problems. In the YSR, the cut-off points of 63 and 60 were used to label the results as “clinically intense disorder” and “borderline.” On average, participants in the incarcerated group reached the clinical threshold for externalizing disorders and for total problems.

Participants in the incarcerated group also scored significantly higher compared to those in the non-incarcerated group on all CTQ scales: emotional abuse (*p* = 0.008), physical abuse (*p* < 0.001), sexual abuse (*p* < 0.001), emotional neglect (*p* = 0.021) and physical neglect (*p* = 0.001). In the unadjusted model, the difference between the two groups was marginal for emotional neglect (*p* = 0.091), but the difference became statistically significant in the adjusted model (*p* = 0.021). The effect size was large for physical abuse, moderate for sexual abuse and physical neglect, and small for emotional abuse and emotional neglect. The CTQ also proposed thresholds to qualify the results as clinically significant. Participants in the incarcerated group reached this threshold for physical and sexual abuse as well as for emotional and physical neglect, but not for emotional abuse. Participants in the non-incarcerated group were consistently below the clinical threshold.

Finally, participants in the incarcerated group had significantly higher scores on all YPI subscales compared to the participants in the non-incarcerated group: lifestyle (*p* < 0.001), interpersonal relationships (*p* = 0.034) and affectivity (*p* < 0.001). The effect size was strong for impulsive dimension seeking, moderate for affective dimension and low for interpersonal dimension.

### Changes in Mental Health Problems in the Incarcerated Group

Table 4 presents the evolution in the incarcerated groups' mental health problems at the end of the stay for the three YSR global scales. The score for externalized problems significantly decreased over time (*p* = 0.017) whereas internalized problems and total problems remained similar. The score for externalized problems decreased from 71.32 to 67.93, but remained within the clinical zone. The score for externalized problems at the end of the stay remained higher compared to that of the non-incarcerated group (*p* < 0.001, not reported in Table 4).

**Table 4:** Changes in self-assessment of psychological problems during the stay in incarcerated group (*n* = 54).

**Global scales youth self report**	**Pre-test measurement**	**Post-test measurement**	**Unadjusted ***p***-value^a^**	**Adjusted ***p***-value^b^**
Externalized problems	71.32	67.93	**0.030**	**0.017**
Internalized problems	53.02	53.59	0.100	0.068
Total problems	63.22	60.65	0.480	0.362

*Internalized problems: total anxiety/depression, withdrawal/depression, somatic complaints*.

*Externalized problems: total violation of rules, aggressive behavior*.

*Total problems: total of the internalized, externalized, attention, social and thinking problem scales*.

a *p-value of the mixed linear model (pre-post time measured in number of days)*.

b *p-value of the linear mixed model controlling for age, gender, socio-economic level and number of days before inclusion in the study (pre-post time measured in number of days)*.

*Highlighted in bold: statistically significant differences at the 0.05 cut-off between the two groups*.

## Discussion

This study showed several essential characteristics of the mental health problems and psychiatric care of youths placed in custody in Geneva, Switzerland.

First, the results confirmed the extremely high prevalence rates of psychiatric disorders, with 82.6% of participants having at least one diagnosed psychiatric disorder. This result echoes the high values of prevalence rates identified in previous studies (40–90%) (5, 7–10) and was consistent with the prevalence rates identified in retrospective studies already conducted in this detention center (88%) (4, 6). Therefore, psychiatric care is crucial to improve detained youths' rehabilitation (20, 21). Unsurprisingly, the most prevalent psychiatric disorders were externalizing disorders, particularly conduct disorders and substance use disorders (11).

The comparison with the non-incarcerated group confirmed that self-reported psychological problems among incarcerated youths were more frequent compared to those reported in the general population. This was true also for trauma and psychopathy traits, but not for internalized disorders (28, 39, 40). However, the incarcerated groups' psychopathy traits do not predict future offending and thus should not yet be used for risk assessment purposes in applied forensic settings (41). Previous studies showed that an intensive treatment program was associated with relatively slower and lower rates of recidivism (14). In addition, female offenders with high psychopathic traits are more likely to have histories of psychiatric hospitalization, and a stronger relation between mental health needs and psychopathic traits compared with male offenders (42).

Deficit in cognitive capacity among incarcerated youths was higher compared to among the non-incarcerated group, especially for the vocabulary and information subtests. Previous studies reported similar findings. Consequently, incarcerated youths with deficit in cognitive capacity may be at high risk of reoffending and developing further behavioral problems (32, 33).

Thus, our sample of incarcerated youths was characterized by intrinsic vulnerabilities (psychiatric disorders, externalized problems and psychopathic traits) and environmental factors (socioeconomic origins, being victims of violence and abuse) (21).

Another key finding was related to psychiatric comorbidities. Psychiatric disorders were very prevalent in this population, but young people also often suffered from several disorders simultaneously. Two-thirds of the participants had a diagnosis of two or more psychiatric disorders. Criminology research has often neglected comorbidities (43, 44). However, previous studies indicate that the presence of comorbid psychiatric disorders leads to a poorer prognosis: early onset of the disorders, more severe symptomatology, less effective treatments and a higher risk of relapse (45–47). From a clinical point of view, screening for psychiatric disorders and comorbidities should be undertaken systematically on the basis of clinical interviews, anamnestic elements and screening scales adapted to this highly vulnerable population. In addition, primary and secondary prevention programs that mainly focus on addiction and violence problems should be promoted.

Regarding health care, most participants (79.1%) had psychiatric care prior to detention. Contrary to our expectations, our results showed that psychiatric care was not associated with the presence of comorbid disorders, even though we could assume that the presence of comorbidities indicated the severity of mental health problems. Conversely, the planned care after detention was associated with psychiatric comorbidities, with care being more likely planned for those with comorbidities. This is in line with the need for integrative mental health care for this population.

Despite this unfavorable clinical picture, the stay in the detention center was associated with a positive change of mental health. Indeed, the domain in which these participants had the highest scores, externalized disorders, was significantly reduced at the end of their stay. This might be due to the intensive medical and educational care provided during the stay, which is similar for all youths. This group of youths in conflict with the law requires adapted and intensive care. Some studies reveal that very few youths receive psychiatric care before admission in a detention center (14, 15), although therapeutic and educational interventions are effective in reducing the risk of recidivism (19, 21). Intensive and interdisciplinary care beneficially affects the short-term evolution, criminological trajectory and mental health of youths in conflict with the law (48, 49). Substance use disorders (58%) and alcohol use disorder (30.4%) among the incarcerated group were highly prevalent. Considering the rapid reduction of psychological distress during the first 3 months of abstinence from a substance (50), this can contribute to the positive change of mental health during the detention stay, which had a median time of 188 days. However, longitudinal follow-up is needed to assess the long-term effect of mental care on youths who have spent time in detention.

This study has a number of limitations, including methodological problems. The first was that the incarcerated group was rather small (*n* = 89) and that only a subsample of these participants completed both the K-SADS-PL (*n* = 69) and the follow-up assessment (*n* = 54). The large dropout rate of the follow-up assessment was due to the difficulty of foreseeing the youths' release that the judicial authorities decided and to assess them before their departure. However, dropouts and completers did not significantly differ in sociodemographic and clinical variables at baseline. Second, we did not have the participation rate since 2011 because it has not been documented. However, the participation rate for 2018–2019 was 70%. Third, the non-incarcerated group was selected using a convenient sampling strategy. Other studies should be based on representative samples of the population in order to have a control group to which incarcerated youths could be compared. Other limitations relate to the instruments the study used. The K-SADS-PL provides information on the presence of a psychiatric disorder, but not on its severity. Beyond the presence of disorders and comorbidities, information that is more detailed on the severity of the disorders would provide a better understanding of this population's problems and needs. Finally, some psychiatric disorders were not considered in the study, such as psychosis and severe intellectual deficit. Youths with severe psychotic symptoms or severe intellectual deficit are usually not placed in custody but referred to a psychiatric hospital. In addition, in the early development of psychosis, patients often have unspecific symptoms likely to lead to under-diagnose psychosis (51). Given these limitations, our findings should be interpreted cautiously and further longitudinal studies are needed.

However, to our knowledge, this is the only prospective study with a longitudinal follow-up focusing on young offenders in Switzerland and a comparison group. This study thus allowed us to identify this vulnerable population's characteristics and needs.

Our findings showed that youths in conflict with the law are characterized by (1) their internal vulnerabilities: a high prevalence of psychiatric disorders (82.6%) and comorbidities (66.7%), lower cognitive functions, mainly at the level of vocabulary and knowledge, externalized problems and psychopathic traits, especially in the impulsive dimension; (2) environmental risk factors: low socio-economic background, victims of violence and sexual abuse; and (3) their psychiatric history: 79.1% received psychiatric care before admission. Besides, the evolution of the most prevalent issues was favorable over time and questions the usual position of the deleterious effect of detention.

## Data Availability Statement

The raw data supporting the conclusions of this article will be made available by the authors, without undue reservation.

## Ethics Statement

The studies involving human participants were reviewed and approved by Cantonal Ethics Research Committee of Geneva (No. 2010-10-240). Written informed consent to participate in this study was provided by the participants' legal guardian/next of kin.

## Author Contributions

PH, SB, LM, and MD designed the study. SB and LM collected data. SB performed statistical analyses. PH drafted the manuscript. PH, LM, DB, ML, LJ, MD, HW, and SB substantively revised it. All authors approved the submitted version of the manuscript and agreed both to be personally accountable for the author's own contributions and to ensure that questions related to the accuracy or integrity of any part of the work and contributed to the study conception and data interpretation.

## Conflict of Interest

The authors declare that the research was conducted in the absence of any commercial or financial relationships that could be construed as a potential conflict of interest.

## Publisher's Note

All claims expressed in this article are solely those of the authors and do not necessarily represent those of their affiliated organizations, or those of the publisher, the editors and the reviewers. Any product that may be evaluated in this article, or claim that may be made by its manufacturer, is not guaranteed or endorsed by the publisher.
